# Diet, gonadal sex, and sex chromosome complement influence white adipose tissue miRNA expression

**DOI:** 10.1186/s12864-017-3484-1

**Published:** 2017-01-17

**Authors:** Jenny C. Link, Yehudit Hasin-Brumshtein, Rita M. Cantor, Xuqi Chen, Arthur P. Arnold, Aldons J. Lusis, Karen Reue

**Affiliations:** 1 Department of Human Genetics, David Geffen School of Medicine, University of California, Los Angeles, CA USA; 2 Department of Medicine, David Geffen School of Medicine, University of California, 90095 Los Angeles, CA USA; 3 Department of Integrative Biology and Physiology, University of California, 90095 Los Angeles, CA USA; 4 Laboratory of Neuroendocrinology of the Brain Research Institute, University of California, 90095 Los Angeles, CA USA; 5 Molecular Biology Institute, University of California, 90095 Los Angeles, CA USA

**Keywords:** microRNA, Adipose tissue, Sex differences, Sex chromosome

## Abstract

**Background:**

MicroRNAs (miRNAs) are small non-coding RNA molecules that regulate gene expression by targeting specific mRNA species for degradation or interfering with translation. Specific miRNAs are key regulators of adipogenesis, and are expressed at different levels in adipose tissue from lean and obese mice. The degree of lipid accumulation and distribution of white adipose tissue differs between males and females, and it is unknown whether sex differences in adipose tissue-specific miRNA expression may contribute to this dimorphism. Typically, sex differences are attributed to hormones secreted from ovaries or testes. However, the sex chromosome complement (XX *versus* XY) is also a determinant of sex differences and may regulate miRNA expression in adipocytes.

**Results:**

To identify sex differences in adipose tissue miRNA expression and to understand the underlying mechanisms, we performed high-throughput miRNA sequencing in gonadal fat depots of the Four Core Genotypes mouse model. This model, which consists of XX female, XX male, XY female, and XY male mice, allowed us to assess independent effects of gonadal type (male *vs*. female) and sex chromosome complement (XX *vs*. XY) on miRNA expression profiles. We have also assessed the effects of a high fat diet on sex differences in adipose tissue miRNA profiles. We identified a male–female effect on the overall miRNA expression profile in mice fed a chow diet, with a bias toward higher expression in male compared to female gonadal adipose tissue. This sex bias disappeared after gonadectomy, suggesting that circulating levels of gonadal secretions modulate the miRNA expression profile. After 16 weeks of high fat diet, the miRNA expression distribution was shifted toward higher expression in XY *vs*. XX adipose tissue. Principal component analysis revealed that high fat diet has a substantial effect on miRNA profile variance, while gonadal secretions and sex chromosome complement each have milder effects.

**Conclusions:**

Our results demonstrate that the overall miRNA expression profile in adipose tissue is influenced by gonadal hormones and the sex chromosome complement, and that expression profiles change in response to gonadectomy and high fat diet. Differential miRNA expression profiles may contribute to sex differences in adipose tissue gene expression, adipose tissue development, and diet-induced obesity.

**Electronic supplementary material:**

The online version of this article (doi:10.1186/s12864-017-3484-1) contains supplementary material, which is available to authorized users.

## Background

MicroRNAs (miRNAs) are small noncoding RNA molecules that modulate gene expression by targeting mRNA transcripts for degradation or interfering with mRNA translation [1, 2]. These 21- to 22-nucleotide molecules are derived from the processing of longer mRNAs transcribed from intergenic regions or introns of protein-coding genes [3, 4]. Approximately 2000 miRNA genes have been recognized in the human and mouse genomes, many of which are expressed in a tissue-dependent manner [5, 6]. Bioinformatic predictions estimate that 30–80% of mammalian mRNAs are targeted by miRNAs, and a given mRNA may be targeted by multiple miRNAs [7, 8]. In addition, a single miRNA can regulate multiple mRNA transcripts, potentially orchestrating coordinate regulation of several genes within a metabolic pathway [9, 10]. It is generally thought that miRNAs act to fine-tune mRNA levels, but there is also evidence of full repression of specific protein production in some cases [11–13].

miRNAs have important roles in the regulation of metabolic homeostasis. For example, miR-33a and miR-33b, which are embedded within genes for sterol regulatory element-binding proteins *Srebf2* and *Srebf1*, respectively, have key roles in the modulation of cholesterol homeostasis [14, 15]. The discovery of the cellular roles of miR-33a/b in the repression of genes involved in cellular cholesterol export has revealed a new therapeutic target. Indeed, studies in mice and non-human primates have shown that antagonism of miR-33a/b reduces plasma lipid levels and atherosclerosis [16, 17]. Many other examples of important roles for miRNAs in metabolism have emerged recently (reviewed in 18). Among these are roles for miRNAs in the regulation of adipogenesis [18–20]. Specific miRNAs that enhance adipocyte differentiation (miR-30c, miR-143, miR-146b, and miR-378; [21–24]) or inhibit adipocyte differentiation (miR-27, miR-130, and miR-138; [25–27]) have been identified. Some miRNAs that are induced during adipocyte development are dysregulated in obese mice [28, 29]. While a few specific miRNAs have been well characterized, the roles of the majority of miRNAs expressed in adipose tissue are unknown.

Many properties of mammalian adipose tissue accumulation, distribution, and metabolism differ between males and females [30]. To understand the basis for sex differences in fat storage, previous studies have assessed differences in mRNA expression levels in male *vs*. females fat depots [31, 32]. It is likely that sex differences also occur in miRNA expression levels in adipose tissue, and that these differences may contribute to sex differences in mRNA levels. Consistent with this, sex differences in miRNA expression levels have been reported in brain [33], lung [34], and liver [35] and may influence sex-specific disease development and pathogenesis [36]. However, the effect of sex on miRNA expression in adipose tissue has not been investigated.

The mechanisms underlying sex differences in miRNA levels have not been studied. Sex differences in metabolism can be attributed to both hormonal and genetic factors [37, 38]. Gonadal hormones have been considered to be the primary drivers of sex differences, and some miRNA levels in a variety of tissues are responsive to estradiols [39]. Importantly, however, the sex chromosome complement also plays a major role in determining adiposity [40, 41], and it is possible that this is mediated in part by miRNAs. To dissect the contributions of gonadal secretions and sex chromosome complement to sex differences, we have used the Four Core Genotypes (FCG) mouse model. The FCG model generates mice with four combinations of gonads and sex chromosomes: XX female, XX male, XY female, and XY male mice [42]. FCG mice have a Y chromosome that carries a mutation (denoted Y^−^) in the testis-determining gene, *Sry*, such that XY^−^ mice develop ovaries rather than testes. In addition, FCG mice carry an autosomal *Sry* transgene that independently segregates from the Y^−^ chromosome. Mice that inherit the *Sry* transgene develop testes. A cross between XX females and XY^−^ *Sry* males produces four combinations of gonads and sex chromosomes (XX male and female, XY male and female). Using this model, we previously demonstrated that the presence of two X chromosomes leads to increased adiposity compared to XY mice, independently from effects of ovaries or testes [40].

Here, we performed RNA sequencing (RNA-seq) of small RNAs present in gonadal fat from FCG mice to identify sex differences in miRNA expression levels in adipose tissue. We determined that sex hormones and sex chromosomes each influence the miRNA expression profile. In addition, comparison of mice fed chow *vs*. high fat diets revealed sex-specific changes in miRNA profiles in response to diet-induced obesity. Our findings have implications for understanding sex differences in adipose tissue metabolism and the development of diet-induced obesity.

## Methods

### Animals

Four Core Genotypes (FCG) C57BL/6 mice were bred and genotyped as described previously [40, 43]. Briefly, XX female mice were mated with XY^−^(*Sry+*) male mice to generate XX, XX(*Sry+*), XY^−^, and XY^−^(*Sry+*) offspring, and genotyping was performed by PCR to detect the *Sry* transgene and a Y-chromosome–specific sequence. Where indicated, gonadectomy was performed at 75 days of age as described previously [40]. During surgery, gonads were removed while leaving surrounding adipose tissue in place.

Gonadal males and females were housed in separate cages and maintained at 23 °C with a 12:12 h light:dark cycle. Gonadally intact females were analyzed without estrous cycle synchronization, such that gene expression values represent an average over the estrous phases. All mice were initially fed Purina mouse chow diet containing 5% fat (Purina 5001; PMI Nutrition International, St. Louis, MO). Where specified, mice were fed a high fat diet (60% calories from fat, Bio-Serv Diets #S3282, Flemington, NJ) for 16 weeks beginning at 3.5 months of age (4 weeks after gonadectomy). Adipose tissue was harvested from all mice at 7.5 months of age.

Mouse studies were conducted after approval by the Institutional Animal Research Committee of the University of California, Los Angeles.

### RNA extraction and quality control

At the time of dissection, gonadal fat tissue was flash frozen in liquid nitrogen and stored at −80 °C. Small RNAs were isolated from 100 mg tissue samples using QIAzol and Qiagen’s miRNeasy Mini kit (Cat. 217004, Qiagen, Valencia, CA). After homogenization, samples were centrifuged at 12,000 × *g* for 10 min to separate the transparent lipid layer from the pink organic layer. Only the organic layer was used in chloroform extraction. All subsequent steps followed the Qiagen protocol. RNA samples were submitted to Agilent BioAnalyzer Eukaryote Total Nano-RNA chip analysis, yielding RNA integrity numbers of 7.5 or greater.

### miRNA library preparation

The feasibility of using pooled sequencing libraries was assessed by sequencing miRNAs from individual samples separately and after pooling. Indexed libraries were generated from adipose tissue of three XX females fed a high fat diet. Average counts of mapped miRNAs from the individual libraries were compared to miRNA counts of the pooled library using Pearson’s product-moment correlation. For the remaining conditions, three samples of each genotype were pooled into equimolar amounts for library preparation. In total, twelve miRNA libraries were made: libraries for each of the four genotypes in chow-fed, gonadally intact mice, chow-fed, gonadectomized mice, and gonadectomized mice fed a high fat diet.

miRNA libraries were processed individually using a standard protocol from Illumina TruSeq Small RNA kit, with indices 1–12, and gel purified according to manufacturer’s instructions. Final sequencing library concentration (19.07 nM) was determined using KAPA library quantification qPCR kit (KK4854, Kapa Biosystems, Wilmington, MA). Sequencing was performed at the Broad Stem Cell Research Center core facility at UCLA, on Illumina HiSeq 2000.

### Reference sequence determination

miRNA gene expression is typically quantified by counting reads that map to the miRNA genes. However, in some families of miRNAs, several genes give rise to identical mature sequences, thus it is impossible to distinguish which miRNA gene gave rise to the mature sequence based on sequencing alone. We performed reference preprocessing to compile a list of uniquely expressed mouse miRNA sequences, regardless of their gene of origin, so that quantification was done at the level of mature miRNA sequence, rather than at the gene level.

A Reference sequence was compiled based on all mature and precursor miRNA sequences available from the main repository of miRNA studies, miRBase version 18 (http://www.mirbase.org), and included all mature and precursor sequences. The respective mature sequences within precursor sequences were masked to “N”, to prevent mature sequences mapping both to mature and precursor. Thus, reads mapping to precursor sequences mapped to precursors exclusively.

In cases where several genes gave rise to the same mature miRNA sequence, that sequence was represented only once in the Reference under one name, with an additional column listing all matching genes as potential targets in the results. In total, 1007 mature miRNA sequences were unique (Additional file 1), while an additional 256 miRNAs grouped into 82 unique sequences (Additional file 2). In 7 cases, the miRNAs were not completely equivalent, with one miRNA being 1 base shorter than the other. In such cases, the shorter sequence would map to both mature sequences, while the longer one would map uniquely to the longer sequence. For these pairs, the counts of the shorter sequence were determined as the total counts mapping to longer sequence minus the unique mappings to longer sequence (Additional file 3).

### Sequence read processing

Reads were demultiplexed in.qseq format based on perfect match to barcode sequence, and converted to.fastq format using in-house Perl scripts (available upon request). Read quality was assessed with FastQC (http://www.bioinformatics.babraham.ac.uk/projects/fastqc/) and adaptor sequence was clipped using cutadapt tool (https://cutadapt.readthedocs.io). Clean reads 18–30 bp long were retained for subsequent mapping and analysis. Identical reads were collapsed using collapse.pl script from mirDeep package (https://www.mdc-berlin.de/8551903/en/). Reads were then mapped to the Reference with BWA aligner allowing for up to 1 mismatch and no gaps. Using these criteria, 80–90% of the reads mapped to the Reference sequence.

### Read counting and result table

Reads that uniquely mapped to one target were counted towards that target. Reads that had both perfect and imperfect (1 mismatch) matches, were counted only towards the perfect match target. Results were summarized in a count table that listed the counts for each target in each sample (Additional file 4). Precursor miRNA counts are composed of reads mapping exclusively to precursor sequences, and not to any mature miRNA sequence in the Reference. For each Reference sequence, the total read count and the number of reads that originate from 1-mismatch reads were presented separately. The number of mismatched reads is a subset of total reads mapping to the Reference.

Percent abundance of miRNAs was calculated by normalizing the reads from one miRNA to the total number of reads mapped for all miRNAs. The mean of the four genotypes across all diet/gonadectomy conditions is represented in Fig. 2b.

### Quantitative PCR

Select miRNAs were validated with quantitative PCR, using individual samples that comprised the pooled samples that were sequenced. Taqman primers (Cat. 4427975, Thermo Fisher Scientific, Waltham, MA) were used according to manufacturer’s protocols. Briefly, 10 ng RNA was reverse transcribed using TaqMan MicroRNA Reverse Transcription Kit (Cat. 4366596, Thermo Fisher Scientific). Quantitative real-time PCR was performed on Bio-Rad CFX Connect Real-Time PCR Detection System using SsoFast Probes Supermix (Cat. 172–5231, Bio-Rad, Hercules, CA). miRNAs of interest were normalized to two housekeeping genes: U6 small nuclear RNA and small nucleolar RNA (snoRNA) 251. Expression was quantified using ΔCt for miR-221 or standard curve for miR-133a, miR-192, and miR-205. Standard curves were generated using 4 serial dilution points of cDNA combined from all biological samples.

### miRNA target prediction

miRNAs with exclusively male–female or XX–XY log_2_ fold changes greater than 0.5 were selected for target prediction. Targets for miRNAs were predicted by mirdb.org, version 5.0 [44]. All targets with a prediction score of 95 or above were included in subsequent functional analysis using DAVID, version 6.7 [45, 46]. The functional annotation tool was used to identify enriched annotation terms in the KEGG Pathway. Unique KEGG pathways with a Bonferroni-corrected *p*-value < 0.05 were included in Additional file 5: Table S1.

### Statistics

Pearson’s product-moment correlation, exact binomial tests, Wilcoxon rank sum tests, and principal component analyses were performed using R and visualized with the R packages ggplot2 and ggbiplot [47–49]. Because each genotype and diet condition was measured in one pooled sample from each genotype/diet/sex condition, the present analysis did not have adequate power to assess the significance of treatment and genotypic effects on expression levels of individual miRNAs. Instead, we viewed each miRNA as an independent contributor to the overall miRNA expression profile of each genotype under each experimental condition, and we report patterns of expression among groups.

In qPCR experiments, groups were compared using two-way ANOVA (NCSS 2001; Number Cruncher Statistical Systems, Kaysville, UT), with gonadal sex and sex chromosome complement as independent factors. Values for miR-192 and miR-205 were log_10_-transformed before statistical analysis.

## Results

### Study design to identify sex differences in adipose tissue miRNAs under three physiological conditions

**Fig. 1:**
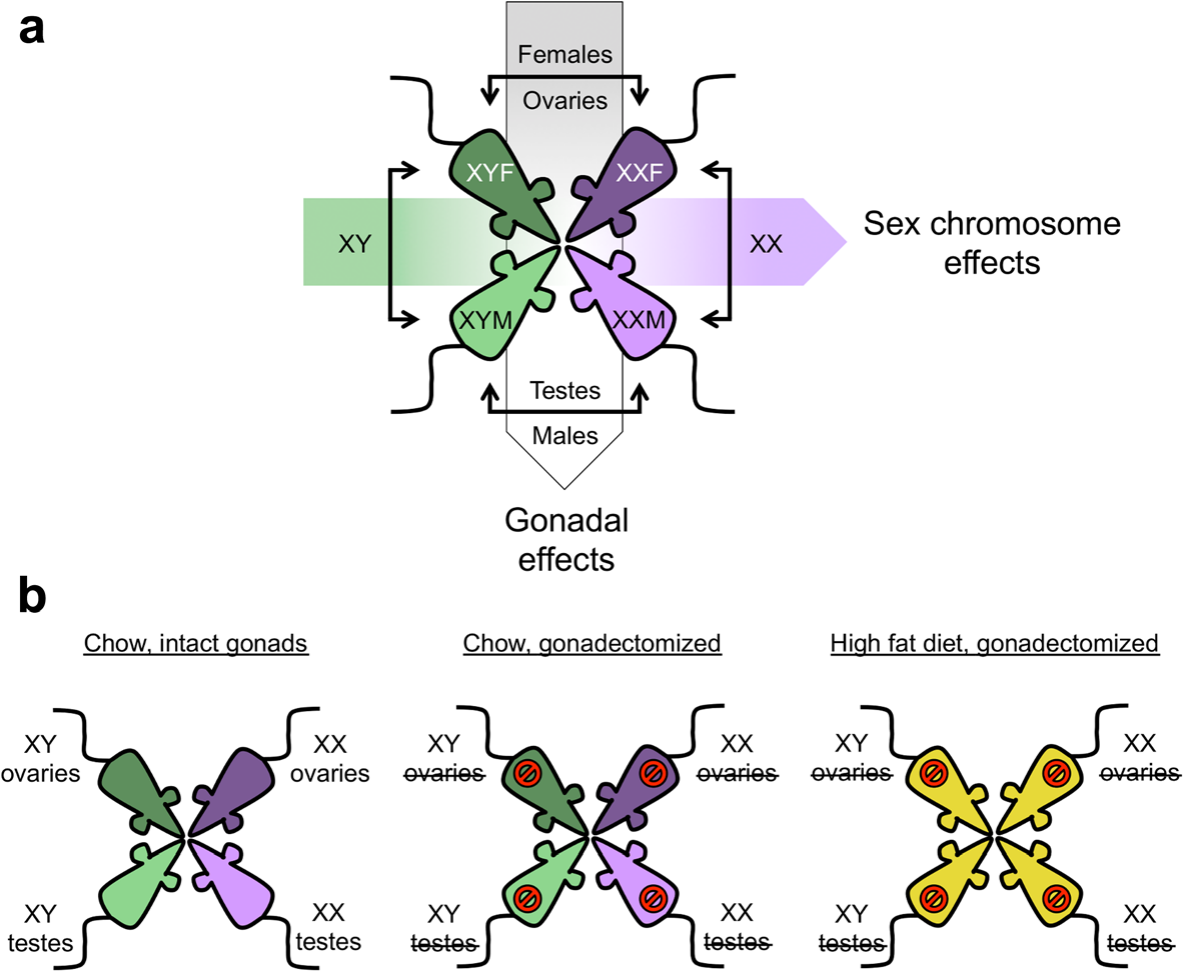
Study design to identify sex and dietary effects on miRNA expression. **a** miRNAs were sequenced in adipose tissue from the Four Core Genotypes mouse model (comprising XX female, XX male, XY female and XY male mice). A comparison of miRNA levels in female XX and XY adipose tissue with those in male XX and XY tissue allows detection of effects due to gonadal type. A comparison of miRNA levels in female and male XX adipose tissue with those on female and male XY tissue allows detection of effects due to sex chromosome type. **b** To determine the effects of acute gonadal hormones of diet, miRNA sequencing was performed in Four Core Genotypes cohorts that were fed a chow diet in the gonadally intact state, fed a chow diet and gonadectomized as adults to remove the acute effects of gonadal secretions, and in mice that were gonadectomized as adults and fed a high fat diet for 16 weeks

To assess the effect of gonadal hormones and sex chromosomes on miRNA levels in adipose tissue, we sequenced miRNAs in the gonadal fat depot of FCG mice. The four genotypes of this model allow analysis of sex differences based on the presence of male *vs*. female gonads (by comparing male XX and XY with female XX and XY mice), or the presence of XX *vs*. XY chromosome complement (by comparing XX female and male with XY female and male mice) (Fig. 1a). Additionally, we analyzed the effect of circulating gonadal hormones by comparing changes in miRNA profiles in mice with intact gonads *vs*. mice after gonadectomy (Fig. 1b). Finally, we assessed the effect on adipose tissue miRNA profile of high fat diet, and its interaction with sex, by comparing mice on chow and high fat diets (Fig. 1b). Adipose tissue samples from the gonadal fat depot were collected from all cohorts at the same age (7.5 months). Sequenced and mapped miRNAs were counted to establish miRNA expression profiles.

### Sex differences in adipose tissue miRNA profiles

**Fig. 2:**
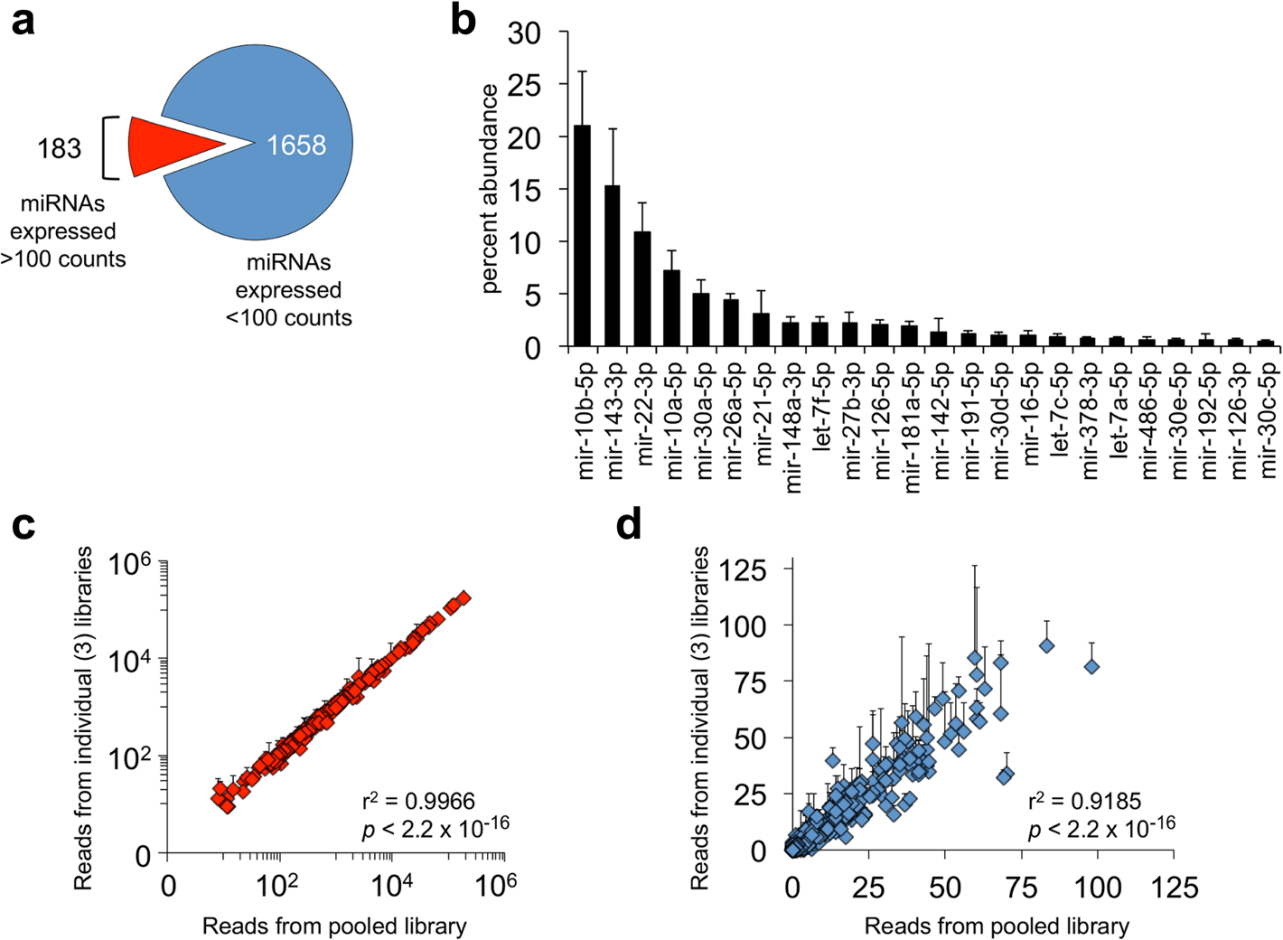
Highly expressed miRNAs in gonadal fat. **a** Out of 1841 mapped miRNAs, 183 were expressed at over 100 counts per million reads in at least one of the twelve libraries. These miRNAs are termed “highly expressed” miRNAs. **b** Percent abundance of miRNAs in gonadal fat of FCG mice. Values represent mean ± SD of the twelve sequencing libraries. **c**, **d** Three individual miRNA sequencing libraries representing biological replicates of XX females fed a high fat diet are correlated with the pooled sequencing library composed of those same mice. Highly expressed gonadal fat miRNAs (**c**) are more correlated between the individual and pooled libraries than miRNAs that are expressed at a lower level (**d**). Values represent mean ± SD. r^2^ and *p*-values were calculated using Pearson product–moment correlation

We sequenced and mapped 1,841 mature and precursor miRNAs in gonadal adipose tissue of FCG mice. We identified 183 miRNAs (10%) that were expressed at substantial levels in gonadal fat (>100 counts per million reads in at least one sample type) (Fig. 2a). Three miRNA species—miR-10b-5p, miR-143-3p, and miR-22-3p—accounted for nearly 50% of all miRNA reads (Fig. 2b). An additional 21 miRNA species each accounted for at least 0.5% of the gonadal adipose tissue miRNA pool. 17 of the 24 (70.8%) most prevalent miRNAs in mouse adipose tissue have previously been shown to be abundant in human subcutaneous white adipose tissue [50].

To streamline our sequencing process for the twelve conditions shown in Fig. 1, we assessed the feasibility of pooling three biological replicates for miRNA sequencing for each condition. We sequenced samples from three individual mice (from XX female mice fed high fat diet) separately and after pooling together. We found that the average miRNA reads of the three individual samples were highly correlated with the values from the pooled sample (Fig. 2c, d; *p* < 2.2 × 10^−16^; *r* ^2^ = 0.9966 for the 183 most abundant miRNAs, *r* ^2^ = 0.9185 for the lower abundance miRNAs). Based on these findings, we concluded that pooled libraries were a satisfactory representation of individual biological samples. For analyses described below, we focused on the 183 most abundantly expressed adipose tissue miRNAs, as these are most likely to have physiological significance.

**Fig. 3:**
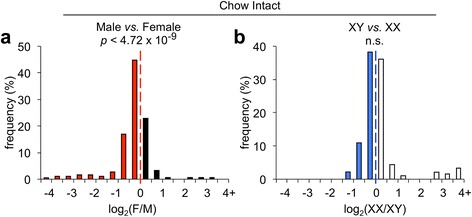
Distribution of miRNAs in chow-fed gonadally intact mice. Distribution of female-to-male ratios (**a**) and XX-to-XY ratios (**b**) in highly expressed miRNAs. Ratios were calculated for each miRNA, log_2_-transformed, and binned in 0.5 increments. Values less than −4 were grouped into the first bin, and values greater than 4 were grouped into the last bin. *Red bars* refer to male (M)-biased miRNAs, *black bars* refer to female (F)-biased miRNAs, *blue bars* refer to XY-biased miRNAs, and *white bars* refer to XX-biased miRNAs. *p*-values were calculated using the exact binomial test

To establish a baseline profile of sex effects on miRNA expression in adipose tissue, we sequenced miRNAs from mice fed a standard chow diet. We first asked whether there was a male or female bias in overall miRNA expression by examining the ratio of female-to-male miRNA levels (“female” values were the average of female XX and XY miRNA read counts, and “male” values were the average of male XX and XY counts). Female-to-male ratios of each miRNA were log_2_-transformed and plotted as a histogram (Fig. 3a). Adipose tissue miRNAs had a significant bias toward higher expression in male compared to female gonadal adipose tissue (exact binomial test, *p* < 4.72 × 10^−9^). In the chow-fed cohort, there was no significant bias in miRNA distribution between XX and XY mice, although we cannot exclude the possibility that the sex chromosome complement may alter expression levels of individual miRNA species. Thus, in the basal physiological condition of chow-fed animals, the presence of male *vs*. female gonads led to a significant difference in adipose tissue miRNA expression profile.

### Adipose tissue miRNAs regulated by gonadal hormones

We hypothesized that male–female differences in miRNA expression profile were caused by differences in gonadal hormones. Gonadal hormones cause sex differences in two primary ways. First, they impose permanent, or “organizational,” effects during development, such as the effect of testosterone on male genital differentiation. Second, male and female gonadal hormones in the circulation have acute/reversible effects on processes such as gene expression. To investigate the effects of acute gonadal hormone action on miRNA expression, we gonadectomized adult FCG mice (at 10 weeks of age) and assessed miRNA profiles 5 months later, during which time circulating gonadal hormones were absent. With this experimental design, male–female differences that disappear after gonadectomy can be attributed to acute hormone effects.

**Fig. 4:**
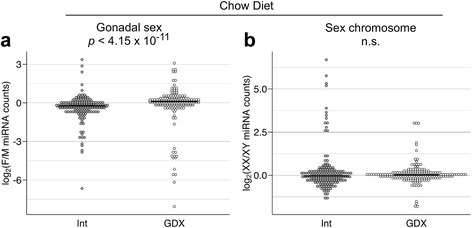
Gonadectomy alters distribution of miRNAs in chow-fed mice. Distribution of female-to-male ratios (**a**) and XX-to-XY ratios (**b**) in highly expressed miRNAs. Ratios were calculated for each miRNA and log_2_-transformed in gonadally intact (Int) and gonadectomized (GDX) FCG mice fed a chow diet. Data for gonadally intact (Int) mice are identical to Fig. 3a–b, but binned in 0.1 increments and compared to data distribution for GDX mice. Each dot represents one of the 183 highly expressed miRNAs. *Black bars* represent median values. Distributions were significantly different for female-to-male ratios, but not for XX-to-XY ratios. *p*-values were calculated using the Wilcoxon rank sum test. F, female; M, male

In gonadectomized mice, the miRNA profile expressed as female-to-male ratio exhibited a small bias toward higher miRNA expression levels in females compared to males (exact binomial test, *p* < 0.02). Comparison of female–male ratio distributions between gonadectomized mice and gonadally intact mice revealed a significant difference (Wilcoxon rank sum test, *p* < 4.15 × 10^−11^). This represented a shift from a profile with male > female expression ratios in gonadally intact mice to a profile with a slight bias toward female > male ratios in gonadectomized mice (Fig. 4a; median value for log-transformed female-to-male ratios: −0.24 for intact and 0.095 for gonadectomized mice). The miRNA ratio distribution of XX *vs*. XY mice was similar in intact and gonadectomized mice (Fig. 4b). Taken together, these data suggest that acute effects of gonadal secretions, but not sex chromosome complement, produce sex biases in overall miRNA levels in mice fed a chow diet. We note, however, that there are subsets of miRNAs with extreme values in female-to-male ratios and in XX-to-XY ratios in gonadectomized mice, suggesting that, in the absence of circulating gonadal hormones, sex and sex chromosome differences remain in individual miRNAs.

### Adipose tissue miRNA sex differences influenced by high fat diet or obesity

Several studies have shown that miRNA expression levels are regulated during adipogenesis or are altered in obesity [28, 51]. However, it is unknown whether sex influences miRNA expression levels in obese adipose tissue. To identify sex and/or sex chromosome factors that alter miRNA expression in obesity apart from the acute effect of gonadal hormones, we performed miRNA-seq on gonadal fat from FCG mice that were gonadectomized (10 weeks of age) and then fed a high fat diet (60% calories from fat) beginning 1 month later for 4 months.

**Fig. 5:**
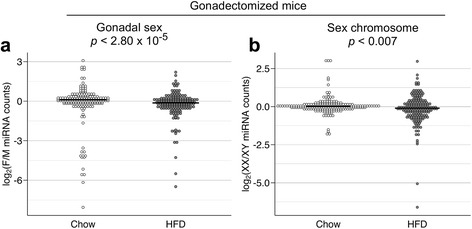
High fat diet alters miRNA expression levels in a sex-dependent and sex chromosome complement-dependent manner. Distribution of female-to-male ratios (**a**) and XX-to-XY ratios (**b**) in highly expressed miRNAs. Ratios were calculated for each miRNA and log_2_-transformed in gonadectomized (GDX) FCG mice fed a high fat diet (HFD). Data for chow-fed gonadectomized mice are identical to Fig. 4a–b. Distributions were significantly different for female-to-male ratios and for XX-to-XY ratios. *p*-values were calculated using the Wilcoxon rank sum test. F, female; M, male

The distribution of female-to-male miRNA read count ratios was significantly male-biased (exact binomial test, *p* < 0.003). This male bias in gonadectomized high fat diet-fed mice (median = −0.132) represents a significant shift in miRNA expression compared to the distribution in chow-fed mice that had been gonadectomized at the same age and therefore represents an effect of diet (Wilcoxon rank sum test, *p* < 2.80 × 10^−5^; Fig. 5a). Interestingly, miRNA expression levels were also biased toward the XY sex chromosome complement in mice fed a high fat diet (exact binomial test, *p* < 0.02). This indicates that the sex chromosome complement, in addition to gonadal hormones, influenced miRNA expression in mice fed a high fat diet. Comparison of the distributions of XX-to-XY ratios indicated a shift from a slight XX bias in chow-fed mice (median XX/XY ratio = 0.021) to an XY bias in fat-fed mice (median XX/XY ratio = −0.100; Wilcoxon rank sum test, *p* < 0.007; Fig. 5b). In addition, there are a number of miRNAs that shift to the extreme ends of the sex chromosome distribution in mice fed a high fat diet. These data suggest that, in gonadectomized mice, high fat diet and obesity modulate miRNA expression in a sex- and sex chromosome-specific manner.

We followed up on select miRNAs that had distinct sex-influenced expression patterns and/or were previously implicated in adipocyte biology. Quantitative real-time PCR (qPCR) of miR-133a-3p, previously shown to inhibit brown adipocyte differentiation [52], was upregulated in XX compared to XY mice in the chow-fed, gonadally intact group (Additional file 5: Figure S1A). This difference was abolished after gonadectomy, suggesting an interplay between gonadal hormones and sex chromosome complement. miR-192-5p levels were higher in males compared to females in gonad-intact mice, but this sex difference is lost after gonadectomy, and an XX > XY difference emerges (Additional file 5: Figure S1B). Conversely, miR-205-5p showed a sex-chromosome bias (XX > XY) in gonadally intact mice and a sex bias (M > F) in gonadectomized mice fed a chow diet (Additional file 5: Figure S1C). However, when mice were fed a high fat diet, miR-192-5p and miR-205-5p showed no sex or sex chromosome differences. miR-221-3p, which is located on the X chromosome and has been previously associated with obesity and adipogenesis [28], showed no sex or sex chromosome difference in mice fed a chow diet (Additional file 5: Figure S1D). However, mice fed high fat diet showed increased levels of miR-221-3p in XX male and female mice compared to XY mice. These miRNA species illustrate several distinct patterns of expression that are dependent on gonadal type, gonadal hormones, diet, and sex chromosome complement, and emphasize that sex differences exist in miRNAs that have been studied previously in the context of adipose tissue and obesity.

**Fig. 6:**
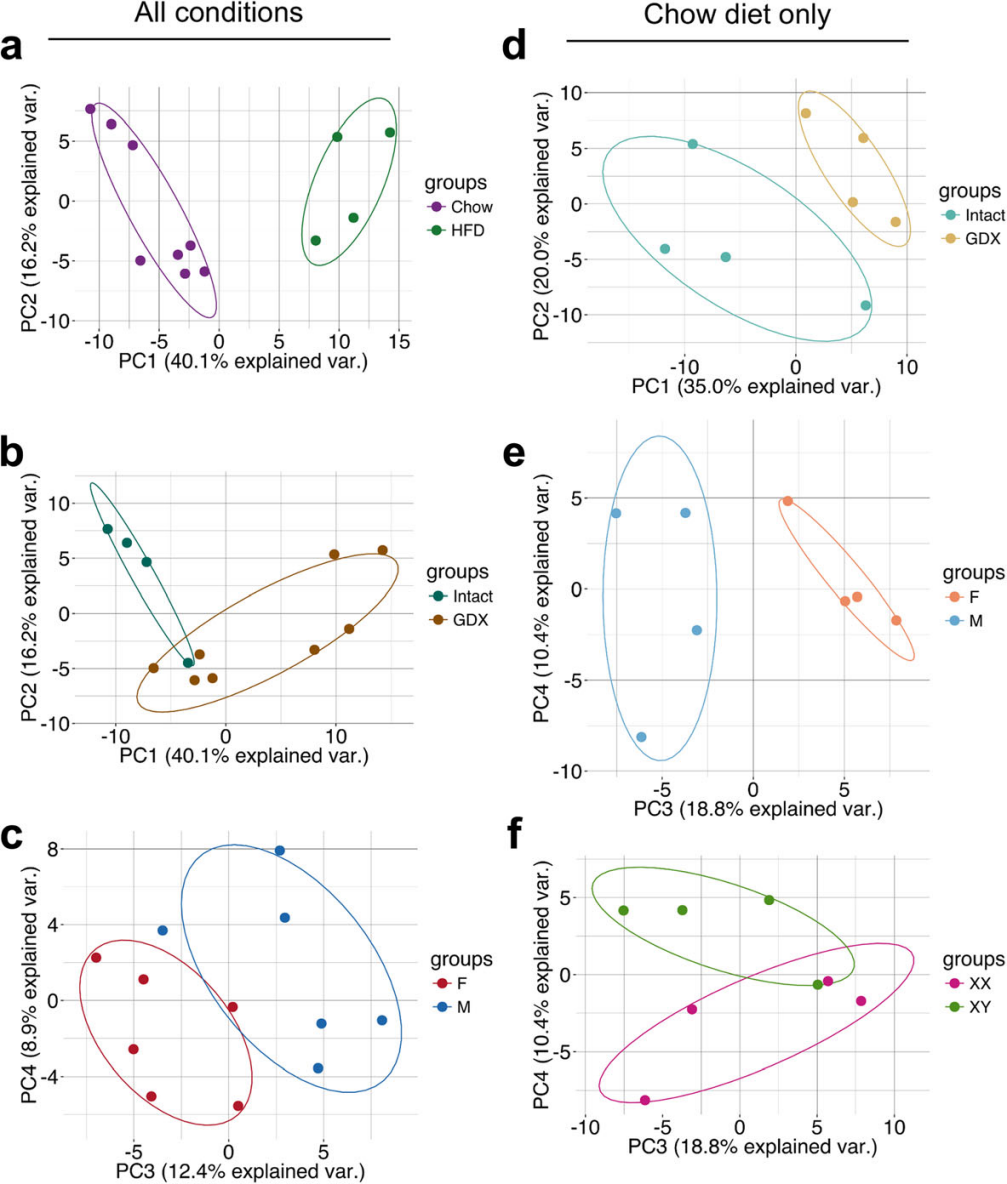
Principal component analysis (PCA) reveals factors influencing miRNA correlation covariance. PCA of all twelve sequencing libraries (**a**–**c**) or chow-fed samples only (**d**–**f**). Each dot represents a sequencing library and consists of normalized read counts for each of the 183 highly expressed miRNAs. Dots are colored and encircled according to diet (**a**), gonadal state (**b**, **d**), gonadal sex (**c**, **e**), or sex chromosome complement (**f**). HFD, high fat diet; Int, gonadally intact; GDX, gonadectomized; F, female; M, male

To examine the relationship between diet, acute hormone effects, gonadal type, and sex chromosome complement effects on miRNA expression, we performed principal component analysis. Diet distinguished miRNA expression levels to the greatest degree, explaining 40.1% of miRNA covariance (Fig. 6a). Gonadal state (gonadally intact *vs.* gonadectomized) and gonadal type (male testes *vs.* female ovaries) led to a milder but detectable separation (Fig. 6b–c). The sex chromosome complement did not lead to divergence of miRNA covariance. Because diet had such a pronounced effect on miRNA covariance, we performed principal component analysis on mice fed only the chow diet to determine if sex-biasing factors still influenced miRNA covariance. The first two components revealed deviation of gonadally intact mice from gonadectomized mice (Fig. 6d). In addition, when examining the third and fourth principal components, females clustered separately from males, and XX mice exhibited a slight degree of separation from XY mice (Fig. 6e–f). We note that the effect of the sex chromosome complement was detectable after removing the overwhelming effect of diet. Taken together, these data suggest that diet is a major factor in miRNA expression levels, and that sex factors, such as acute gonadal secretions, male–female gonads, and XX–XY chromosome complement, also play important roles in modulating miRNA levels.

## Discussion

miRNAs are key regulators of gene expression through their effects on the degradation or translation of mRNA transcripts. In metabolic tissues, miRNAs influence gene expression related to adipogenesis, cholesterol homeostasis, and glucose homeostasis [18]. These metabolic processes are also affected by sex. While there is evidence for sexual dimorphism in miRNA expression in brain, skeletal muscle, and immune cells [39], sex effects on miRNAs in adipose tissue have not been characterized. In this study, we used the FCG mouse model to identify sex chromosome complement and gonadal hormone effects on miRNA abundance in gonadal fat. We sequenced miRNAs in three distinct cohorts of FCG mice in different hormonal and dietary conditions to determine the effects of circulating gonadal hormones and high fat diet on sex differences in gonadal fat miRNAs. We selected the gonadal fat depot for our studies because it has been widely studied in the mouse obesity field. However, it is clear that differences exist between gonadal fat and other visceral fat depots, as well as subcutaneous fat depots, and it would ultimately be of interest to delineate sex differences across these distinct depots. Our analyses focused on the most abundant miRNAs, as we suspect that sex differences in their levels are the most likely to have significant physiological effects.

By comparing overall miRNA profiles across diet, gonadal state, gonadal type, and sex chromosome complement, we determined that each of these factors influence miRNA expression in white adipose tissue. In gonadally intact mice, we identified a male bias in miRNA expression levels and no significant bias between XX and XY mice. As expected, the distribution of male–female differences in the levels of miRNAs was altered by gonadectomy, suggesting that circulating gonadal hormones regulate miRNA levels and are responsible for some male–female sex differences. Gonadectomy also influences adiposity [53], which may impact miRNA expression. We have previously shown that gonadectomized XX mice have increased body weight and adiposity compared to gonadectomized XY mice [40]. Because we studied mice 5 months after gonadectomy, we cannot distinguish whether changes in miRNA expression preceded increases in adiposity or resulted from them. However, male–female differences in miRNA levels do not correlate with XX–XY differences in body weight, suggesting that sex differences in adiposity do not determine sex differences in overall miRNA levels. Studies measuring miRNA levels after gonadectomy but before weight gain will be required to address these questions.

High fat diet feeding alters sex and sex chromosome differences in miRNA levels, suggesting a diet–sex interaction in miRNA expression. It is notable that the observed sex and sex chromosome biases result from changes in overall miRNA distributions and not from changes in a few specific miRNAs. High fat feeding revealed a sex chromosome bias in miRNA expression ratios that was not apparent in chow-fed animals. This raises the possibility that differential miRNA expression levels may contribute to the dramatic difference in adiposity of XX compared to XY mice in response to high fat diet [40]. Thus, mice with two X chromosomes gain more fat than XY mice, and this difference is enhanced upon feeding a high fat diet [40]. Further studies will be required to elucidate the physiological roles of individual sex-biased miRNAs in adipogenesis and diet-induced obesity.

Some miRNAs with distinct sex-biased patterns in expression, such as miR-133a and miR-221, have been implicated in adipogenesis [28, 52]. Other miRNAs may have yet unidentified roles in lipid metabolism. An initial analysis of potential target genes for sex-biased miRNAs that we identified revealed enrichment in specific metabolic pathways (Additional file 5: Table S1). These predictions suggest that pathways such as mTOR signaling and phosphatidylinositol signaling may be important in mediating sex differences in healthy and/or diseased adipocytes. Studies to determine the impact of these predicted target genes and corresponding pathways on sex differences in obesity provide a unique area for future research.

## Conclusions

This study represents a first look at mechanisms underlying sex differences in miRNA profiles in adipose tissue. We demonstrate that miRNAs in adipose tissue are influenced by diet, gonadal state, gonadal type, and sex chromosome complement. Principal component analysis revealed that diet is the leading factor in miRNA expression covariance, while gonadal state, gonadal type, and sex chromosome complement were milder but important regulators. By recognizing the innate sex differences in miRNA levels, we can better understand sex differences in fat accumulation, distribution, and adipose pathophysiology.

## Additional files

Additional file 1: 1007 mature miRNA sequences that mapped uniquely to the miRBase 18. (TXT 15 kb)
Additional file 2: 256 miRNAs that mapped to 82 unique sequences. (TXT 5 kb)
Additional file 3: 7 cases of miRNAs that were of unequal length, and thus read counts had to be adjusted for non-unique mapping. (TXT 364)
Additional file 4: Count table summarizing read counts for 1841 mature and precursor miRNA sequences in fifteen sequencing libraries. Perfectly matched counts are normalized to total library reads per million. (XLS 512 kb)
Additional file 5: Figure S1. Expression of select miRNAs with distinct sex- and sex-chromosome-biased patterns, measured by TaqMan qPCR. **Table S1.** Enrichment of specific cellular pathways in predicted targets of sex-influenced miRNAs. (PDF 268 kb)

## Abbreviations

FCG: Four core genotypes
miR, miRNA: microRNA
mRNA: messenger RNA
