# Supplementary material for: Diet, gonadal sex, and sex chromosome complement influence white adipose tissue miRNA expression
Source: BMC Genomics. 2017 Jan 17;18:89. doi: 10.1186/s12864-017-3484-1 (PMC5240420; doi:10.1186/s12864-017-3484-1)
Supplement: Additional file 5: Figure S1. — Expression of select miRNAs with distinct sex- and sex-chromosome-biased patterns, measured by TaqMan qPCR. Table S1. Enrichment of specific cellular pathways in predicted targets of sex-influenced miRNAs. (PDF 268 kb) [file 12864_2017_3484_MOESM5_ESM.pdf]

Figure S1

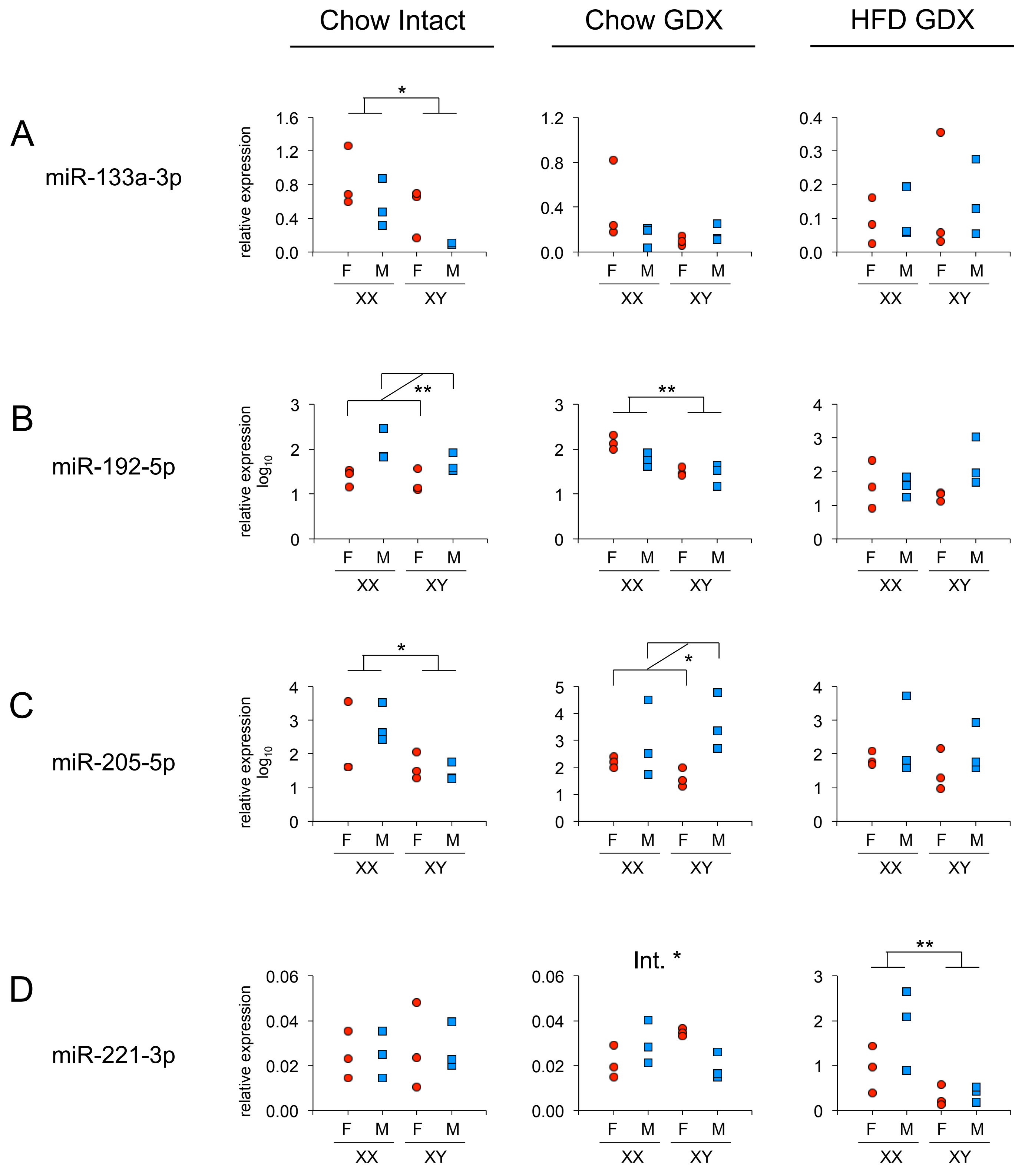

Figure S1. Expression of select miRNAs with distinct sex- and sex-chromosome-biased patterns. Levels of expression for miR-133a-3p (A), miR-192-5p (B), miR-205-5p (C), and miR-221-3p (D) were measured by TaqMan qPCR in chow-fed gonadally intact and gonadectomized mice, and in high fat diet-fed gonadectomized mice. Each point represents an individual mouse. Note that for miR-192-5p and miR-205-5p, values are plotted on logarithmic scale. Groups were compared using two-way ANOVA. HFD, high fat diet; GDX, gonadectomized; F, female; M, male; *, $p<0.05$; **, $p<0.01$.

Table S1. Enrichment of specific pathways in predicted targets of sex-influenced miRNAs

KEGG Pathway	Fold Enrichment	Bonferroni-corrected *P*-value
**Chow-fed, gonadally intact male–female differences**		
mmu04150: mTOR signaling pathway	7.41	0.003
**Chow-fed, gonadally intact XX–XY differences**		
mmu04144: Endocytosis	4.29	0.004
**Chow-fed, gonadectomized male–female differences**		
no significant KEGG pathways		
**Chow-fed, gonadectomized XX–XY differences**		
mmu04120: Ubiquitin mediated proteolysis	7.67	0.03
**High fat diet-fed, gonadectomized male–female differences**		
no significant KEGG pathways		
**High fat diet-fed, gonadectomized XX–XY differences**		
mmu04720: Long-term potentiation	6.19	0.03
mmu04070: Phosphatidylinositol signaling system	5.77	0.05

Table S1.Predicted targets of sex-biased miRNAs were enriched for specific cellular pathways. miRNAs with exclusively male–female or XX–XY $\log_2$ fold changes greater than 0.5 were selected for target prediction. Targets for miRNAs were predicted by mirdb.org, version 5.0. All targets with a prediction score of 95 or above were included in subsequent functional analysis using DAVID, version 6.7. The functional annotation tool was used to identify enriched annotation terms in the KEGG Pathway. Unique KEGG pathways with a Bonferroni-corrected *p*-value < 0.05 were included in this table.
